# Oligodendrocyte-Specific Mechanisms of Myelin Thinning: Implications for Neurodegenerative Diseases

**DOI:** 10.3389/fnins.2021.663053

**Published:** 2021-03-24

**Authors:** Monokesh K. Sen, Md Jakir Hossain

**Affiliations:** ^1^School of Medicine, Western Sydney University, Penrith, NSW, Australia; ^2^Peter Duncan Neuroscience Research Unit, St. Vincent's Centre for Applied Medical Research, Darlinghurst, Sydney, NSW, Australia; ^3^School of Medical Sciences, UNSW Sydney, Sydney, NSW, Australia

**Keywords:** multiple sclerosis, neurodegeneration, unfolded protein response, demyelination, protein aggregation

## Introduction

Myelination is a fundamental molecular event within the vertebrate central nervous system (CNS) where oligodendrocytes wrap around axons to form a myelin sheath which allows rapid “saltatory” conduction of action potentials along the axons (Bercury and Macklin, 2015). Loss of myelin/demyelination leaves a naked axon with a compromised speed of signal conduction, susceptible to inflammatory attack by immune cells that eventually results in axonal degeneration (Simons and Nave, 2016). While demyelination and neurodegeneration are hallmark pathologies of many CNS diseases, including Multiple Sclerosis (MS), myelin thinning was not considered detrimental until recent years. For decades, the myelin sheath, including myelin thickness, was regarded as stable throughout adulthood. In recent years, evidence has been emerging regarding the pathological as well as activity-dependent plasticity in myelin structure, such as progressive myelin thinning in the absence of overt myelin loss or demyelination (Purger et al., 2016; Dutta et al., 2018). However, the molecular mechanisms underlying this process are unclear.

Accumulation of unfolded or misfolded proteins in the endoplasmic reticulum (ER) triggers the unfolded protein response (UPR), which aims or serves to maintain protein homeostasis by multiple mechanisms including attenuation of protein translation and the prevention of translocation of newly synthesized proteins from cytosol to ER (Stone and Lin, 2015). The UPR then leads to the activation of the ER associated degradation (ERAD) of proteins, and when this repair activity is exhausted due to an excess number of unfolded proteins, apoptosis of ER stressed cells occurs (Murao and Nishitoh, 2017). Thus, UPR and ERAD counterbalance each other and cooperate to maintain ER protein homeostasis. UPR plays crucial roles in the development of CNS by affecting neural stem cells, mature neurons and glial cells (Murao and Nishitoh, 2017) and the impairment of UPR has been linked to many CNS diseases including MS (Stone and Lin, 2015). Notably, within the CNS, UPR has differential effects on neurons and glial cells; specifically, oligodendrocytes are known to be more sensitive to disruption of ER homeostasis due to their need to produce large amount of proteins (Murao and Nishitoh, 2017). Therefore, detailed insight into the molecular mechanisms of the UPR specific to oligodendrocytes appears to be important in discovering a new therapeutic strategy for demyelinating diseases.

## Myelin Thinning is Mediated by Sel1L Deficiency in Oligodendrocytes

**Figure 1:**
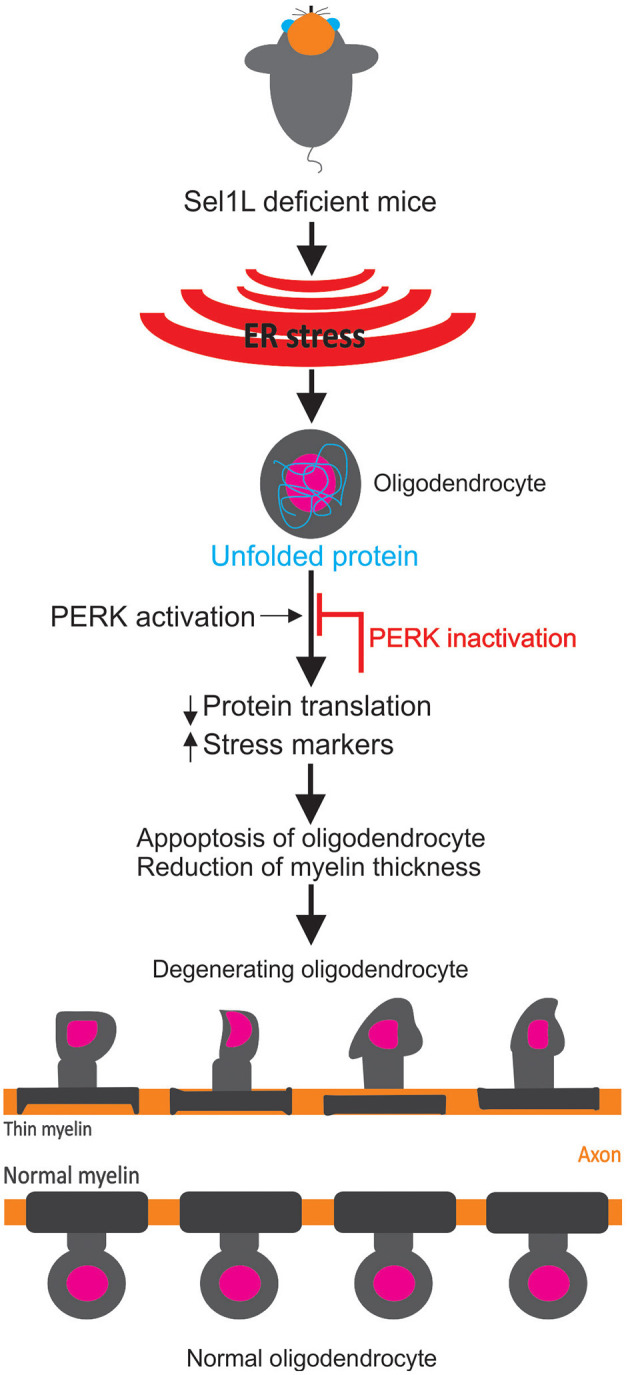
Schematic of the role of the unfolded protein response in myelin structure. Following an endoplasmic reticulum (ER) stress response in Sel1L deficit mice (Suppressor/Enhancer of Lin-12-like), an unfolded protein accumulation inside oligodendrocytes is observed. As a result, the unfolded protein response is activated [as demonstrated by pancreatic ER kinase (PERK) activation and other stress markers] that prevent myelin protein translation. This results in oligodendrocytes degeneration and myelin thinning (Wu et al., 2020). Normal myelin and oligodendrocytes from wild type mice are shown at the bottom.

In a recent study published in *The Journal of Neuroscience*, Wu et al. (2020) provided new evidence regarding how components of the UPR and ERAD work concertedly within oligodendrocytes to maintain myelin thickness in adult mice. This study rigorously analyzed in both male and female mice using multiple experimental approaches including immunohistochemistry, immunoprecipitation, western blot, motor behavior using rotarod and electron microscopy (Wu et al., 2020). Specifically, the authors demonstrated that deficiency of suppressor/Enhancer of Lin-12-like (Sel1L) a key element of ERAD activity, in oligodendrocytes caused ERAD impairment. In addition, activation of the pancreatic ER kinase (PERK) branch of the UPR leads to the inhibition of global myelin protein translation but activates stress-responsive genes [e.g., CCAAT-enhancer-binding protein homologous protein (CHOP)]. These changes resulted in late-onset, progressive myelin thinning in the CNS of Sel1L knockout mice. Subsequently, inactivation of the UPR responses (PERK inactivation) was demonstrated as a salvage pathway to restore myelin protein translation and so reversed the myelin thinning caused by Sel1L deficiency. Notably, there was no difference between male and female mice in the damage caused by Sel1L deficiency. Importantly, the myelin thinning reported by Wu et al. (2020) was attributed to the reduction in myelin protein translation, *not* as a consequence of remyelination or regeneration. Specifically, a myelin protein called proteolipid protein (PLP) was found to be vital in the process of myelin thinning. This was confirmed by showing an exacerbation of myelin thinning, tremoring phenotypes, and a shortened lifespan in Sel1L and PLP double knockout mice compared to Sel1L deficient mice. This confirmed that diminished myelin protein translation was responsible for myelin thinning. They also ruled out the possibility of myelin thinning resulting neither from remyelination, (no myelin loss or oligodendrocyte death), nor from regeneration, (no increase in oligodendrocyte precursor cells or oligodendrocyte proliferation). Importantly, PERK inactivation in oligodendrocytes (Sel1L and PERK double knockout) restored myelin protein translation, reversed myelin thinning, attenuated tremoring phenotypes, prolonged survival, and prevented oligodendrocyte death compared to Sel1L deficient mice. The joint role of UPR and ERAD in myelin thinning as mediated by oligodendrocyte specific deficiency of Sel1L is summarized in Figure 1.

## Effects of Gender, Age, and Duration of Perk Activation in Myelin Thinning in SEL1L Deficient Mice

While Wu et al. (2020) found no difference between male and female mice regarding the effects of Sel1L deficiency on myelin structure, it is known that demyelinating diseases (e.g., MS and Neuromyelitis Optica) disproportionately affects more women than men (Asgari et al., 2011). This suggests an alternating mechanism differing in the male vs. female ratio in these demyelinating diseases. Notably, abnormalities in myelin structure and myelin protein translation were seen in adult mice, but no difference was observed in young mice, suggesting that young mice can compensate for the Sel1L deficiency. Moreover, it is known that both age-dependent differential gene expression and oligodendrocyte maturation play a role in age-related responses to myelination (Bergles and Richardson, 2015), which could partly explain the difference in myelin thinning between young and adult mice. Previous studies have reported both beneficial and detrimental effects of PERK activation on developmental myelination (Stone et al., 2020) via unknown mechanisms. The detrimental effects of PERK activation in adult Sel1L deficient mice reported in this study (Wu et al., 2020) argue against with a previous study that showed PERK activation in adult mice is protective against demyelination (Hussien et al., 2014). Therefore, Wu et al. (2020) hypothesized that short term PERK activation promotes oligodendrocyte survival and acute demyelination whereas long-term PERK activation suppresses myelin protein production and triggers myelin thinning; this needs to be confirmed in future studies.

## Myelin Thinning in SEL1L Deficient Mice Occurred Without Axonal Pathology But Involves Multiple Myelin Proteins

Similar to these findings (Wu et al., 2020), another study showed that myelin thinning and behavioral inflexibility (touch screen based learning tasks) occurred without changes in axon number and diameter, confirming that myelin pathology precedes axonal pathology in demyelinating diseases (Silva et al., 2019). Although the PLP and Sel1L double knockout worsened the detrimental effects on myelin, a reduction of myelin basic protein (MBP) was also observed from 10 weeks of age, resulting in myelin thinning (Wu et al., 2020). Likewise, a previous study reported that abnormal distribution of MBP (rather than total MBP protein levels) contributes to myelin thinning (Silva et al., 2019). This is because in immature oligodendrocytes, MBP is largely localized in the cell body, while in mature oligodendrocytes, MBP is distributed to membranous processes contributing to myelin formation (Silva et al., 2019). These observations (Silva et al., 2019; Wu et al., 2020) suggest that myelin thinning is not exclusive to the loss of a single class of myelin proteins (e.g., PLP), but rather the reduction of multiple myelin proteins (e.g., PLP, MBP) contribute to myelin thinning. Since PERK activation in oligodendrocytes has been reported in many demyelinating diseases (e.g., MS) of the CNS, it poses a challenge for researchers to distinguish between myelin thinning caused by PERK activation and thinner myelin generated by remyelination following demyelination.

## Potential Involvement of Microglia and Astrocytes in the Myelin Thinning Process Caused By SEL1L Deficiency

In contrast to MS and other demyelinating diseases (Luo et al., 2017), the myelin thinning reported here (started at 6 weeks) was independent of microglial activation (seen only at 24 weeks) as there was no myelin breakdown/myelin loss until the later age (Wu et al., 2020). Future studies should address if Sel1L deficiency can also trigger astrocyte activation since it has been shown that astrocytes recruit microglia at the site of demyelination for myelin debris clearance (Skripuletz et al., 2013). However, astrocytes are believed to be less sensitive to UPR disruption (Murao and Nishitoh, 2017), although a recent study showed that the vesicular release of thrombin protease inhibitor from perinodal astrocytes is involved in myelin thinning (by preventing the attachment of myelin to axons) and in reduced conduction speed in optic nerves of mice (Dutta et al., 2018). Whether astrocytes were involved in microglia recruitment in this study (Wu et al., 2020) remains untested. However, the UPR is not unique to MS; rather it is a pathological hallmark of Alzheimer's, Parkinson's, and Prion diseases (García-González et al., 2018), suggesting that these neurodegenerative diseases share similar pathological pathways. In addition, Wu et al. (2020) observed oligodendrocyte degeneration and demyelination without the involvement of adaptive immune cells, as seen in the cuprizone model of demyelination (Sen et al., 2020a). Interestingly, Caprariello et al. (2018) and Almuslehi et al. (2020) showed that oligodendrocyte degeneration and microglial activation recruit adaptive immune cells (pan CD3 T-cells and CD8 T-cells) and trigger subsequent demyelination in cuprizone-fed mice once the blood-brain barrier is compromised (using pertussis toxin injection). Whether oligodendrocytes apoptosis and microglial activation (as shown in this study; Wu et al., 2020) attract adaptive immune cells (e.g., T-cells) into the CNS to facilitate subsequent adaptive immune cell-mediated oligodendrocyte degeneration (Stys et al., 2012; Sen et al., 2020b; as in 'inside-out' theory of MS) remains unexplored.

## Could the PI3K-AKT-MTORC Signaling Axis Regulate Inflammation and Neurodegeneration in SEL1L Deficient Mice?

In addition to the association of PERK activation in myelin thinning in Sel1L deficient mice (Wu et al., 2020), the disruption of PI3K-AKT-mTORC signaling (which is the downstream of the PERK-eIF2α pathway and also involved in protein translation) in oligodendrocyte function and myelination has been demonstrated in many studies (Zhang et al., 2003; Sherman et al., 2012; Lebrun-Julien et al., 2014; Jiang et al., 2016). The kinase Akt (a serine/threonine kinase) expression in oligodendrocytes leads to enhanced myelination in the CNS and this process is linked with the mammalian target of the rapamycin (mTOR) pathway–a mediator of phosphatidylinositol-3 kinase (PI3K)/Akt signaling (Flores et al., 2008). However, the underlying mechanism of the association of mTOR in myelination was unclear until Lebrun-Julien et al. (2014) investigated the effect of functionally relevant subunits (raptor and rictor) of mTOR (mTORC1 and mTORC2) in myelination. They found that conditional deletion of the mTORC1 (but not the mTORC2) gene in the oligodendrocyte progenitor cells prevented their differentiation and maturation, which resulted in reduced production of myelin proteins (MBP, myelin oligodendrocyte glycoprotein, myelin-associated glycoprotein) and thinner myelin (increased g-ratio) in the CNS (Lebrun-Julien et al., 2014). The role of mTOR in preserving myelin structure (prevention from myelin thinness) in this work (Lebrun-Julien et al., 2014) is similar to the role of Sel1L in maintaining myelin thinning as demonstrated by Wu et al. (2020). This suggests that PERK activation in Sel1L deficient mice could also activate downstream mTOR signaling. However, Lebrun-Julien et al. (2014) also showed that tuberous sclerosis 1 (TSC1) ablation-induced over-activation of mTORC1 also caused hypomyelination, suggesting that mTORC1 activity must be balanced and regulated to achieve effective myelination in the CNS. Moreover, the loss of mTOR signaling has also been associated with thinner myelin in the peripheral nerves in mice (Sherman et al., 2012; Figlia et al., 2017). Finally, the dysregulation of the PI3K-AKT-mTORC signaling pathway has been shown in the animal models of MS (EAE and cuprizone) as well as Alzheimer's and Parkinson's (Heras-Sandoval et al., 2014; Giacoppo et al., 2017; Liu et al., 2020). This suggests the involvement of PI3K-AKT-mTORC in neuroinflammation, demyelination and neurodegeneration. Thus, PERK-eIF2α activation in Sel1L deficient mice potentially activates the PI3K-AKT-mTORC pathway downstream to PERK-eIF2α to regulate neuroinflammation, demyelination and neurodegeneration, which should be investigated in future studies in Sel1L deficient mice.

## Perspectives for Future Studies

Since demyelination leads to slowed axonal conduction, electrophysiological recordings in the future can address to what extent myelin thinning in Sel1L knockout mice reduces the speed of conduction in the CNS axons (Mu et al., 2019), which can lead to impaired sensory-motor and/or cognitive functions. Moreover, the lack of motor deficits in young mice (Wu et al., 2020) might be due to a lack of sensitivity in the rotarod test, which reportedly failed to detect subtle motor deficits in some situations, while other tests such as the walking ladder or beam tests were able to detect early motor incoordination (Sen et al., 2020a). Furthermore, proteomic investigation (using a bottom-up approach) of oligodendrocyte progenitor cells from different age groups from Sprague Dawley rats revealed that myelin-associated proteins and proteins associated with inflammatory responses increased with age, while cholesterol-biosynthesis and cell cycle proteins decreased (de la Fuente et al., 2020). Importantly, Wu et al. (2020) suggested that non-myelin proteins might also contribute to the myelin thinning process. Therefore, whether late-onset oligodendrocyte degeneration and myelin thinning depends upon the differential proteome profile in Sel1L knockout mice is worthy of future investigation.

## Conclusion

Taken altogether, this opinion article discussed the landmark work (Wu et al., 2020) that has challenged the long-existed dogma about myelin stability in adulthood, demonstrating that there is room for plasticity in myelin structure. This study reaffirmed the view that oligodendrocytes are more sensitive to ER stress and disruption to UPR than other glial cells. Importantly, this work showed, for the first time, that UPR and ERAD activity join forces in oligodendrocytes to maintain myelin protein translation and myelin thickness. The nature of the late onset, slow progressive myelin thinning following impaired ERAD/UPR is reminiscent of well-known neurodegenerative disease pathologies, including MS. Moreover, this study opens up new approaches to target components of UPR and ERAD activity within the oligodendrocytes as a potential therapeutic strategy for demyelinating diseases.

## Author Contributions

MKS and MJH designed the scope, wrote and edited the manuscript. All authors contributed to the article and approved the submitted version.

## Conflict of Interest

The authors declare that the research was conducted in the absence of any commercial or financial relationships that could be construed as a potential conflict of interest.
